# Safety of ACEI/ARB use in the early (<3 months) post kidney transplant period: a systematic review and meta-analysis

**DOI:** 10.3389/fphar.2024.1522558

**Published:** 2024-12-11

**Authors:** Dahai Fu, Jin Li, Guanglan Zeng, Maozhi Tang

**Affiliations:** ^1^ Department of Nephrology, Wushan County People’s Hospital of Chongqing, Chongqing, China; ^2^ Urinary Nephropathy Center, The Second Affiliated Hospital of Chongqing Medical University, Chongqing, China

**Keywords:** kidney transplant, angiotensin converting enzyme inhibitor, angiotensin receptor blocker, safety, meta-analysis

## Abstract

**Background:**

Data about the safety of ACEI/ARB use in early (<3 months) posttransplant period are restricted and remain controversial.

**Methods:**

This systematic review and meta-analysis included searches of PubMed, Embase and CENTRAL from inception to 31 November 2023, for studies to compare the safety (transplant outcomes and postoperative complications) of ACEI/ARB with non-ACEI/ARB (other antihypertensive medications) initiation in early post kidney transplant period.

**Results:**

Of 1,247 citations identified, 13 eligible studies involving 1919 patients were enrolled for analyses. In short- or long-term observations, there were no differences on pooled serum creatinine between ACEI/ARB and non-ACEI/ARB groups whether initiated within 1 or 1–3 months posttransplant, however, initiation of ACEI/ARB within the first month posttransplant had an advantage effect on the mean creatinine clearance. Early initiation of ACEI/ARB posttransplant reduced the risks of patient death (RR 0.60, *p* = 0.009) and graft loss (RR 0.54, *p* = 0.0002). For postoperative complications, there were no significant differences in acute rejection risk (RR 0.87, *p* = 0.58), delayed graft function risk (RR 1.00, *p* = 0.93), hemoglobin level (MD -0.32 mg/Dl, *p* = 0.46) or urinary protein excretion (MD -0.10 g/24 h, *p* = 0.16) between two groups. However, the ACEI/ARB group had higher incidence of hyperkalemia (RR 2.43, *p* = 0.02).

**Conclusion:**

Early initiation of ACEI/ARB within 3 months posttransplant proved to be basically safe and has renal function recovery benefits, however, hyperkalemia needs to be noted.

## Introduction

Approximately 70%–90% of kidney transplant recipients (KTRs) have either arterial hypertension or require antihypertensive therapy (Miller, 2002; Midtvedt and Hartmann, 2002; Schwenger et al., 2001). Fifty-six percent of them were diagnosed with uncontrolled hypertension (Pisano et al., 2022). Posttransplant hypertension is caused by multifactorial pathogenesis, including pretransplant hypertension, donor hypertension, renin secretion from the native kidney, graft dysfunction, recurrent disease and immunosuppressive treatment, which negatively affects graft and patient survival outcomes. Activated renin-angiotensin-aldosterone system (RAAS) status is ubiquitous in end-stage renal disease (ESRD) patients, while the condition cannot improve well despite kidney transplant (Kovarik et al., 2019; Ohashi et al., 2019; Tiryaki et al., 2018). This provides the advantage of using the RAAS blocker in posttransplant hypertension. At present, RAAS blockers are widely used and are effective in controlling hypertension reducing proteinuria and cardiovascular accidents in CKD/ESRD patients, many studies also have explored beneficial effect of using RAAS blockers posttransplant for blood pressure control, long-term graft function and patient survival. However, the Improving Global Outcomes (KDIGO) and American Society of Transplantation (AST) guidelines are the only guidelines to recommend a target blood pressure in kidney transplant recipients, (Aziz et al., 2018) whereas the safety of early initiation of RAAS blockers and their effect on short-term outcomes have rarely been discussed. More importantly, for fear of early graft arterial stenosis and acute kidney injury, the optimal timing of initiation of RAAS blocker in the early posttransplant period had not reached an agreement, and the safety and effectiveness still has not been well argued. Given the lack of a clear consensus and the limitations of existing analyses, we aimed to conduct a more comprehensive systematic review and meta-analysis to explore the safety of early initiation of RAAS blocker on post kidney transplant outcomes by comparing to other antihypertension agents.

## Material and methods

### Search strategy

This systematic review was conducted according to the Preferred Reporting Items for Systematic Reviews and Meta-Analysis guidelines (PRISMA) (Moher et al., 2010). Two independent investigators (MT and DF) conducted a systematic review of published peer-reviewed research articles by searching PubMed, EMBASE and the Cochrane central register of controlled trials (CENTRAL) databases. The following search terms were used alone or in combination (“Angiotensin-Converting Enzyme Inhibitors” or “ACE-inhibitor” or “renin-angiotensin system” or “angiotensin II receptor blocker” or “renin-angiotensin-aldosterone system” or “ACEI” or “ARB” or “RAAS”) and (“kidney transplant*” or “renal transplant*”). We also reviewed reference lists for additional citations. A Supplementary Material titled “Additional file 1” includes the search strategy.

### Inclusion and exclusion criteria

Studies were eligible for inclusion in the review if they met the following criteria: (1) randomized controlled trial (RCT) or cohort study; (2) kidney transplant patients received ACEI/ARB and compared with patients who received non-ACEI/ARB antihypertensive drugs within 3 months posttransplant; (3) assessed at least one of the following outcomes: creatinine clearance, serum creatinine, 24-h *urine protein excretion*, hyperkalemia, hemoglobin, acute rejection, delayed graft function, patient death and graft loss; (4) patients underwent kidney transplant after 1990; and (5) were English articles only. Studies were excluded if they: (1) were review papers, conference abstracts, theses, news, and nonpeer–reviewed articles; and (2) included those less than 18 years of age (children).

### Outcome

Outcomes were distinguished and analyzed based on whether their follow-up duration was less than 1-year posttransplant (short term) or more than 1-year posttransplant (long term). A “early post kidney transplant period” was commonly referred to within the first 3 months of posttransplant. Safety assessments including: (1) transplant outcomes: creatinine clearance, serum creatinine, patient death and graft loss); (2) postoperative complications: acute rejection, delayed graft function, 24-h *urine protein excretion*, hyperkalemia and hemoglobin.

### Quality assessment

The methodological quality of each eligible trial was assessed independently by two authors (JL and GZ). The Jadad Scale (scored 0–5) was used for RCTs based on three items: assessment of randomization, blinding and description of patient withdrawal and dropout; a score ≥3 indicated good quality (Clark et al., 1999). Additionally, we assessed whether there was allocation concealment and whether an intention-to-treat analysis was performed. Quality assessment of nonrandomized studies was based on the Newcastle–Ottawa Scale (http://www.ohri.ca/programs/clinical_epidemiology/oxford.asp) with the following items: (1) the exposed cohort was truly representative; (2) the cohort was drawn from the same community; (3) ascertainment of exposure; (4) outcome of interest not present at start; (5a) cohorts comparable in age; (5b) cohorts comparable on other factor(s); (6) quality of outcome assessment; (7) follow-up long enough for outcomes to occur; and (8) complete accounting for cohorts. All studies were rated on each indicator (1 star for “yes” and 0 stars for “no”) for a total score between 0 and 9 stars. A study was considered high quality if it was awarded ≥7 stars. Discrepancies in the literature search, data extraction and quality assessment were resolved by discussion and consultation.

### Data extraction

Two authors (DF and JL) extracted the information independently with a standard data extraction table. The following items were extracted: first author and published year, region, study design, sample size, donor and recipient age, initiation time of RASS blocker, the follow-up duration and posttransplant outcome.

### Statistical analysis

RevMan (Version 5.4, Copenhagen: The Nordic Cochrane Centre, The Cochrane Collaboration) was utilized for the execution of the meta-analysis. Dichotomous outcomes were expressed as risk ratios (RRs) with 95% confidence intervals (CIs), and continuous variables were expressed as the mean differences (MDs) with 95% CIs. P < 0.05 was considered statistically significant. Meta-analysis was conducted using the Mantel–Haenszel fixed effect model in the absence of heterogeneity; otherwise, the random effect was applied. Heterogeneity was quantified via the Cochrane Q (P < 0.1) and I (Midtvedt and Hartmann, 2002) statistics [I (Midtvedt and Hartmann, 2002) >50%]. Publication bias and possible sources of heterogeneity were not explored because of the limited literature. We calculated the mean change from baseline and differences if they were not provided in the articles, following the Cochrane handbook for systemic reviews of intervention (Version 5.1.0). Standard deviation (SD) was extracted from the articles or calculated with given standard error (SE). For one study without standard deviation or standard error available, we imputed SD from all the other studies (Furukawa et al., 2006). Log [risk ratio] and SE were calculated by using the calculator provided by RevMan (Version 5.4).

## Results

### Description of eligible studies

**FIGURE 1:**
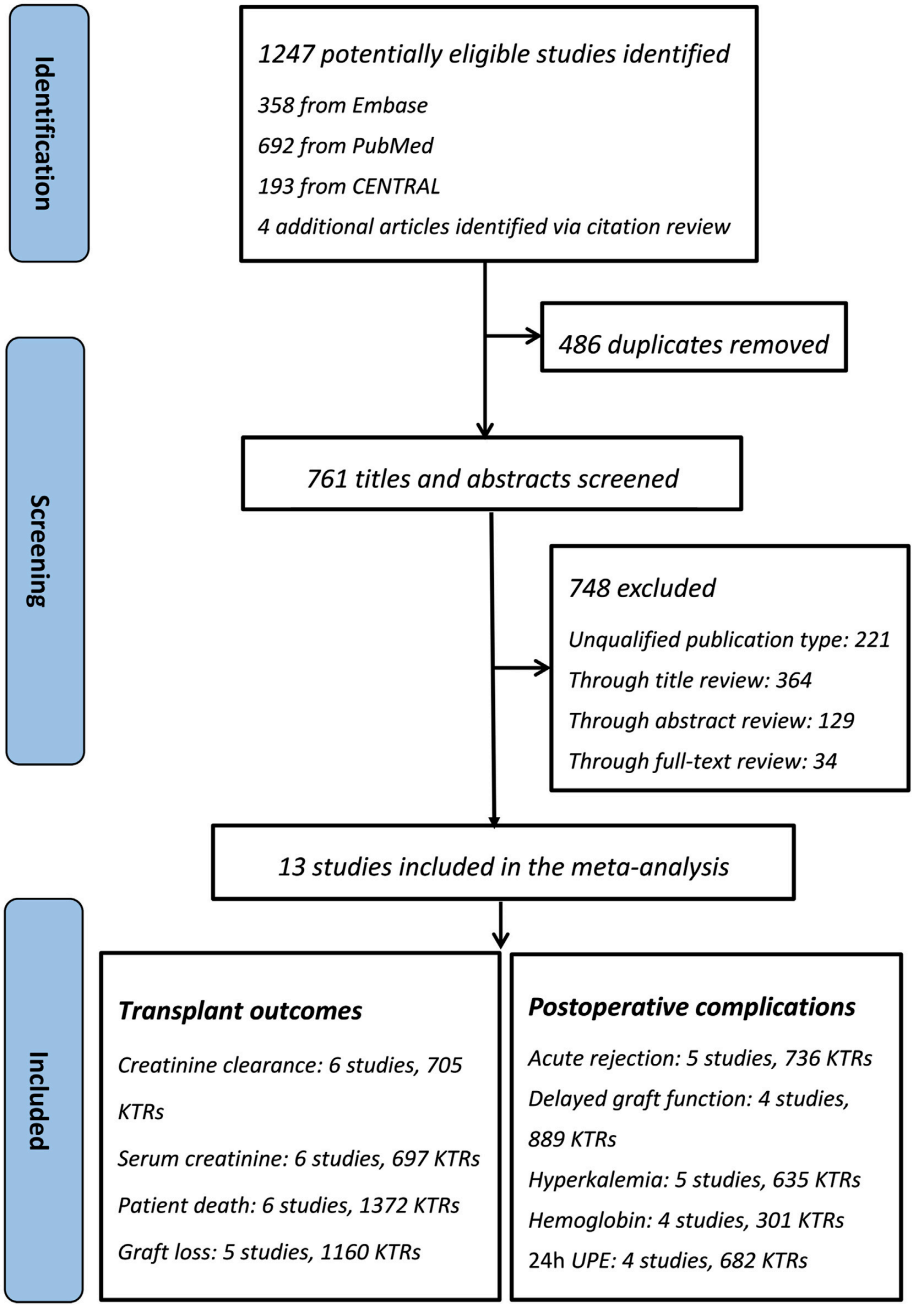
Flow chart for literature search and study selection.

The flow of the included studies is shown in Figure 1. Of the 1,247 citations identified (1,243 via database searches and 4 via secondary searches), 13 relevant studies were identified as eligible for systematic review (Figure 1) (Formica et al., 2006; Hausberg et al., 1999; Glicklich et al., 2011; Formica et al., 2004; Zhang et al., 2013; Suwelack et al., 2003; Chatzikyrkou et al., 2017; Midtvedt et al., 2001a; Lorenz et al., 2004; Ibrahim et al., 2013; Heinze et al., 2006; Midtvedt et al., 2001b; Cockfield et al., 2019). Thirteen eligible studies included 8 RCTs and 5 retrospective cohort studies, whose characteristics are summarized in Table 1. The patient sample sizes ranged from 29 to 436, and the follow-up durations were from 80 days to 10 years. Studies ID 8 and ID 12 were from the same trial but reported different outcomes. The age distributions of the included KTRs between the two groups were not different. The quality of the included studies is displayed in Table 1. The eight RCTs were scored from 3–5 and were all considered good quality trials by using the Jadad Scale measurement. The remaining five retrospective cohort analyses received stars ranging from 6 to 8, and 4 of them were considered good quality studies by using the Newcastle–Ottawa Scale measurement.

**TABLE 1:** Summary of the 13 studies included.

ID	First author	Published year/Location	Study type	Sample size	Antihypertensive intervention		Initiation time of ACEI/ARB after KT	Follow-up	Outcomes	Quality assessment	
				Case/Control	Case	Control				Jadad (scores)	Newcastle-Ottawa (stars)
1	Formica et al. (2006)	2006/United States	RCT	29/27	ARB	CCB	0-30d	12 months	Serum creatinine; hemoglobin; hyperkalaemia	3	
2	Hausberg et al. (1999)	1999/Germany	RCT	35/35	Quinapril	Atenolol	6–12w	24 months	Serum creatinine; creatinine clearance; 24UPE	3	
3	Glicklich et al. (2011)	2011/United States	RCT	14/15	ACEI	non-ACEI/ARB	in 30d	6 months	Serum creatinine; hyperkalaemia	3	
4	Formica et al. (2004)	2004/United States	Retrospective cohort analysis	17/19	ACEI/ARB	CCB	<90d	9 months	Creatinine clearance; hemoglobin		6
5	Zhang et al. (2013)	2019/United States	Retrospective cohort analysis	40/54	ACEI/ARB	non-ACEI/ARB	During hospitalization of KT	1,3,5,7 years	Serum creatinine; creatinine clearance; DGF; AR; patient death; graft loss		7
6	Suwelack et al. (2003)	2003/Germany	RCT	48/48	ACEI	β-blocker	<6–12w	1.5 years	Serum creatinine; creatinine clearance; hemoglobin; 24UPE	4	
7	Chatzikyrkou et al. (2017)	2017/Germany	Retrospective cohort analysis	142/114	ACEI/ARB	non-ACEI/ARB	POD 8	5.4 years	DGF; 24UPE; graft loss		7
8	Midtvedt et al. (2001a)	2001/Norway	RCT	76/78	Lisinopril	Nifedipine	<3w	1.2 years	Serum creatinine; AR; patient death	4	
9	Lorenz et al. (2004)	2004/Austria	Retrospective cohort analysis	116/144	ACEI/ARB	non-ACEI/ARB	POD 1	80d	Serum creatinine; DGF; 24UPE		7
10	Ibrahim et al. (2013)	2013/United States	RCT	77/76	Losartan	non-ACEI/ARB	<3m	5 years	AR; patient death; graft loss; hyperkalaemia	5	
11	Heinze et al. (2006)	2006/Austria	Retrospective cohort analysis	181/255	ACEI/ARB	non-ACEI/ARB	POD 1	2.10 years	Graft loss		8
12	Midtvedt et al. (2001b)	2001/Norway	RCT	54/69	Lisinopril	Nifedipine	<3w	12 months	Hyperkalaemia; hemoglobin	4	
13	Cockfield et al. (2019)	2019/Canada	RCT	142/137	ACEI/ARB	non-ACEI/ARB	POD 1	24 months	DGF, AR, death, graft loss, hyperkalaemia	5	

Abbreviations: RCT, randomized controlled trials; KT, kidney transplantation; UPE, urinary protein excretion; DGF, delayed graft function; AR, acute rejection; POD, post-transplant day. Experimental (ACEI/ARB) group of study ID, 4 included 7 ACEI KTRs, and 10 ARB KTRs, which were separated compared with the control group.

### Safety assessments of transplant outcomes

#### Creatinine clearance and serum creatinine

**FIGURE 2:**
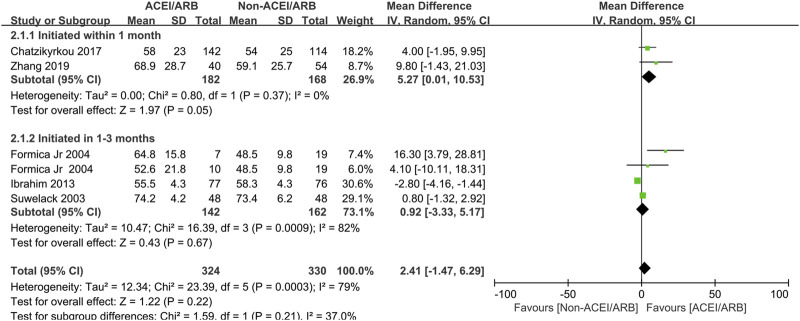
Forest plots of mean creatinine clearance differences between the ACEI/ARB and non-ACEI/ARB groups in short term observation. ACEI/ARB initiated within the first month posttransplant (2.1.1) and at 1–3 months posttransplant (2.1.2). CI, confidence interval.

**FIGURE 3:**
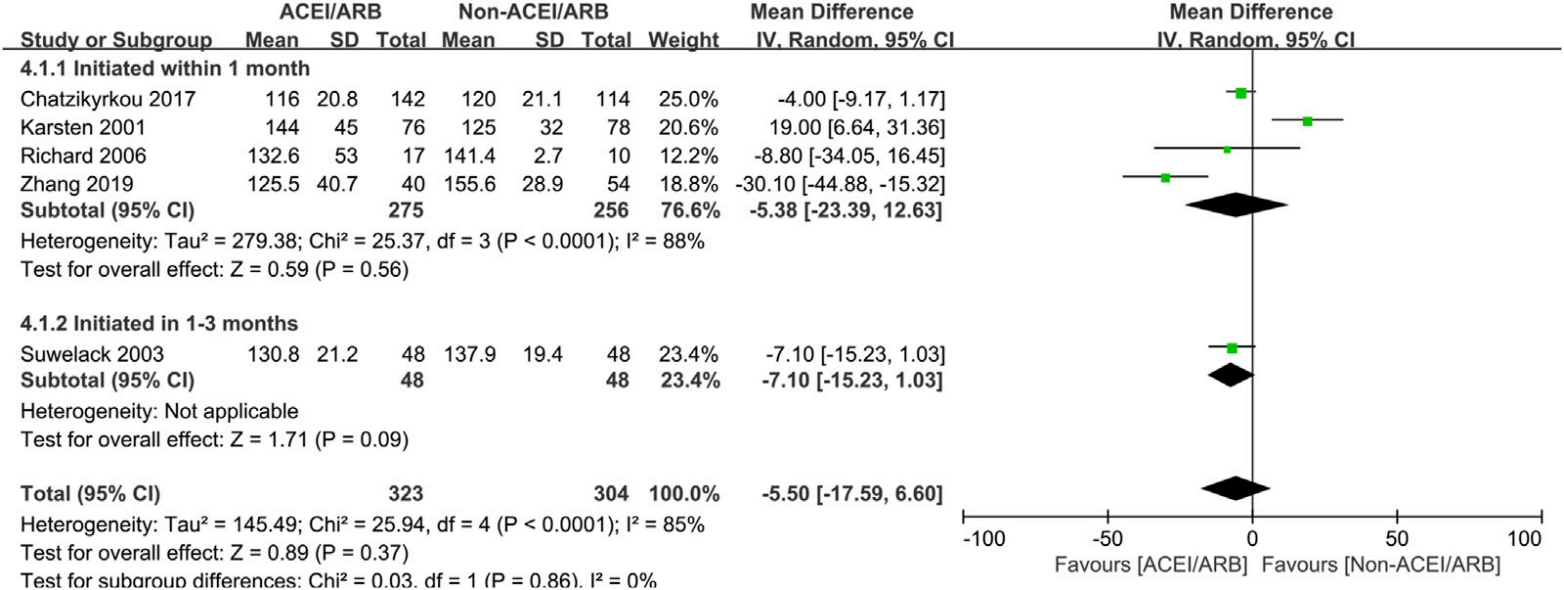
Forest plots of mean serum creatinine differences between the ACEI/ARB and non-ACEI/ARB groups in short term observation. ACEI/ARB initiated within the first month posttransplant (4.1.1) and at 1–3 months posttransplant (4.1.2). CI, confidence interval.

In short term observation, the pooled mean creatinine clearance difference between ACEI/ARB and non-ACEI/ARB groups was not significant (MD 2.41 mL/min; 95% CI -1.47 to 6.29, *p* = 0.22; I^2^ = 79%) (Figure 2), however, further subgroup analysis found that initiation of ACEI/ARB within the first month posttransplant had an advantage effect on the mean creatinine clearance (MD 5.27 mL/min; 95% CI 0.01 to 10.53, *p* = 0.05; I^2^ = 0%) (Figure 2 2.1.1). For outcome of serum creatinine, the pooled mean serum creatinine difference was −5.50 μmol/L (95% CI -17.59 to 6.60, *p* = 0.37; I^2^ = 85%) (Figure 3), referring to no significant difference between two groups, moreover, subgroup analysis found that initiation of ACEI/ARB within the first month posttransplant did not increase the serum creatinine level (MD -5.38 μmol/L; 95% CI -23.39 to 12.63, *p* = 0.56; I^2^ = 88%) (Figure 3 4.1.1).

**FIGURE 4:**
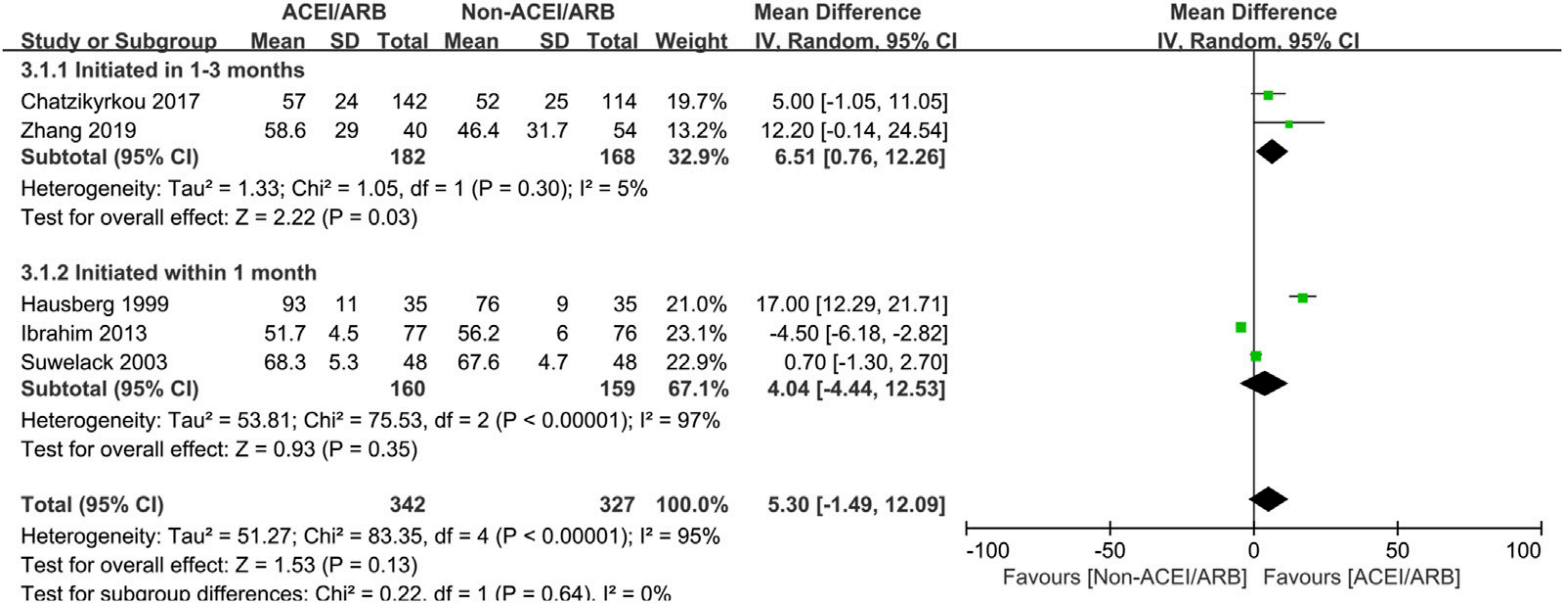
Forest plots of mean creatinine clearance differences between the ACEI/ARB and non-ACEI/ARB groups in long term observation. ACEI/ARB initiated within the first month posttransplant (3.1.1) and at 1–3 months posttransplant (3.1.2). CI, confidence interval.

**FIGURE 5:**
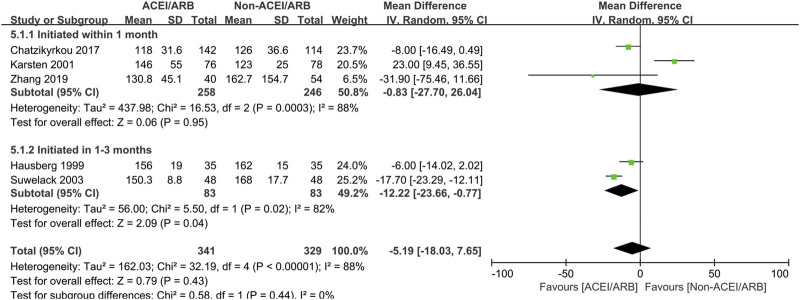
Forest plots of mean serum creatinine differences between the ACEI/ARB and non-ACEI/ARB groups in long term observation. ACEI/ARB initiated within the first month posttransplant (5.1.1) and at 1–3 months posttransplant (5.1.2). CI, confidence interval.

In long term observation, the pooled mean creatinine clearance difference between two groups was also not significant (MD 5.30 mL/min; 95% CI -1.49 to 12.09, *p* = 0.13; I^2^ = 95%) (Figure 4), however, further subgroup analysis found that initiation of ACEI/ARB within the first month posttransplant had an advantage effect on the mean creatinine clearance when compared to non-ACEI/ARB group (MD 6.51 mL/min; 95% CI 0.76 to 12.26, *p* = 0.03; I^2^ = 5%) (Figure 4 3.1.1). For outcome of serum creatinine, the pooled mean serum creatinine difference was not significant between two groups (MD -5.19 μmol/L; 95% CI -18.03 to 7.65, *p* = 0.43; I^2^ = 88%) (Figure 5), however, patient who receive ACEI/ARB at 1–3 months posttransplant had significantly lower serum creatinine level (MD -12.22 μmol/L; 95% CI -23.66 to −0.77, *p* = 0.04; I^2^ = 82%) (Figure 5 5.1.2).

### Patient death and graft loss

Compared to non-ACEI/ARB patients, pooled long-term patient death and graft loss were analyzed. The results demonstrated that early initiation of ACEI/ARB significantly reduced the risks of patient death (RR 0.60; 95% CI 0.41 to 0.88, *p* = 0.009; I^2^ = 0%) (Supplementary Figure 1) and graft loss (RR 0.54; 95% CI 0.40 to 0.75, *p* = 0.0002; I^2^ = 0%) (Supplementary Figure 2).

### Postoperative complications

**FIGURE 6:**
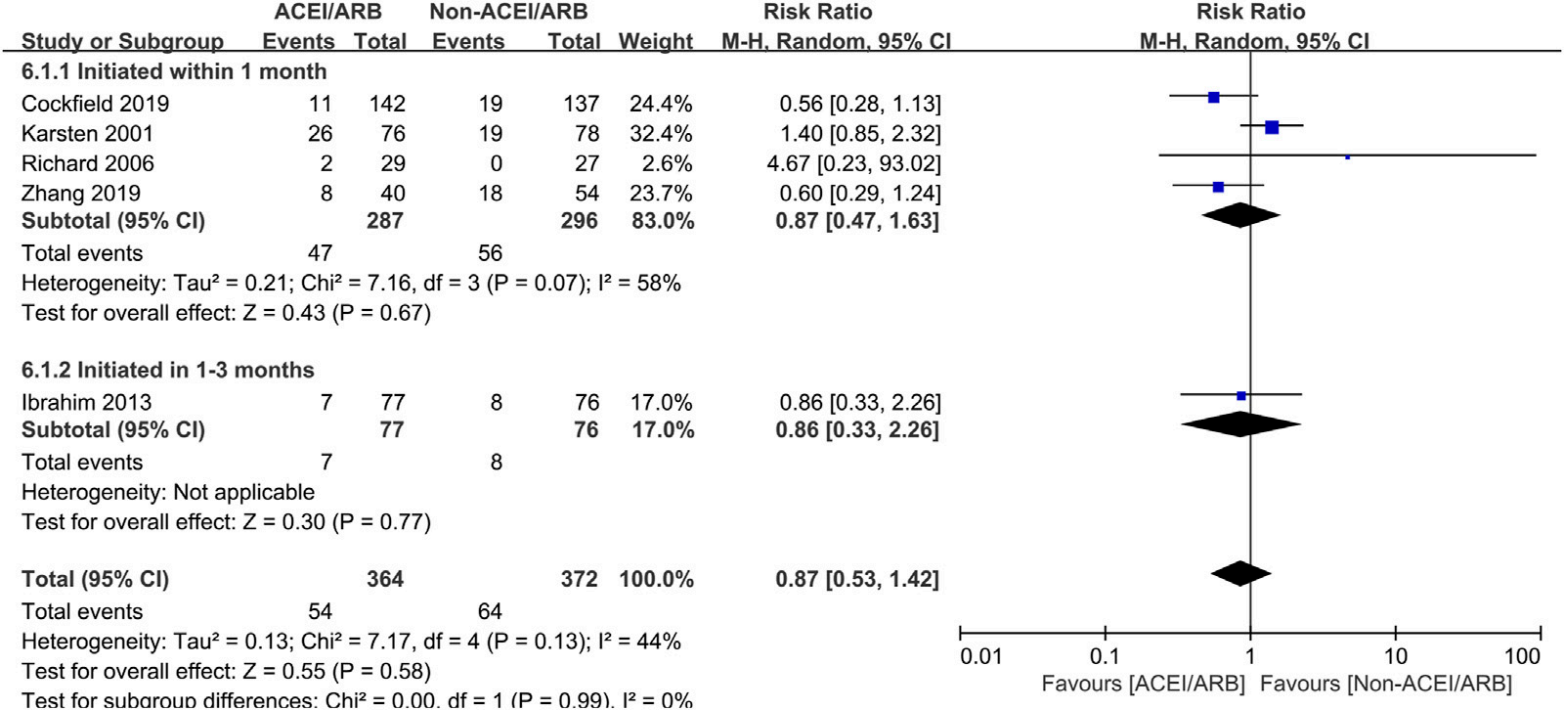
Forest plots depicting the risk ratios of acute rejection (AR) of early initiation of ACEI/ARB versus non-ACEI/ARB groups. ACEI/ARB initiated within the first month posttransplant (6.1.1) and at 1–3 months posttransplant (6.1.2). CI, confidence interval.

**FIGURE 7:**
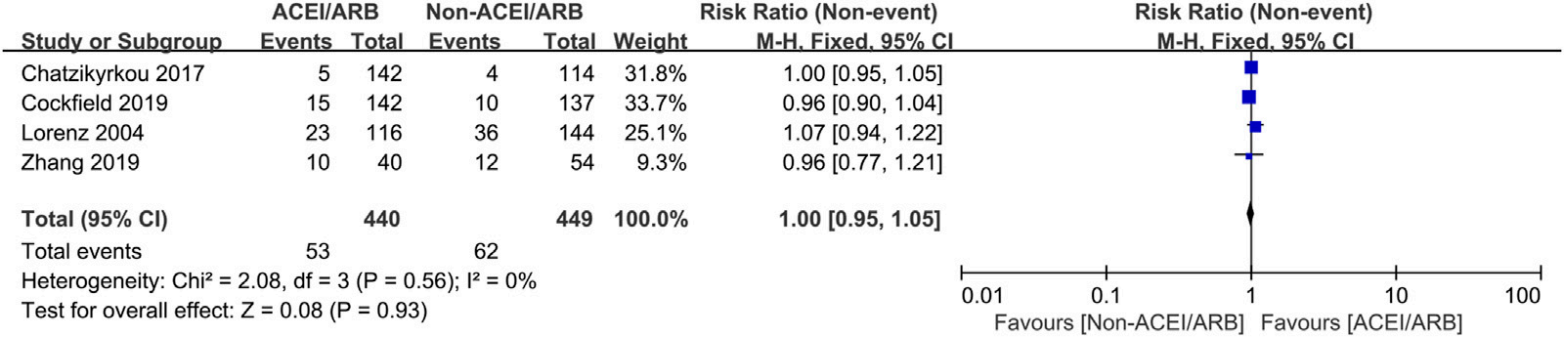
Forest plots depicting the risk ratios of delayed graft function (DGF) of early initiation of ACEI/ARB versus non-ACEI/ARB groups. CI, confidence interval.

Five studies with 736 KTRs estimated AR risk, and 4 studies with 889 KTRs estimated DGF risk. We found that there were no significant differences in AR risk (RR 0.87; 95% CI 0.53 to 1.42, *p* = 0.58; I^2^ = 44%) (Figure 6) and DGF risk (RR 1.00; 95% CI 0.95 to 1.05, *p* = 0.93; I^2^ = 0%) (Figure 7) between two groups. Typically, we also found that initiation of ACEI/ARB within the first month posttransplant did not increase AR risk (RR 0.87; 95% CI 0.47 to 1.63, *p* = 0.67; I^2^ = 58%) (Figure 6 6.1.1). For other complications, compared to non-ACEI/ARB group, ACEI/ARB group had no significantly differences in hemoglobin level (MD -0.32 mg/dL; 95% CI -1.16 to 0.52, *p* = 0.46; I^2^ = 84%) (Supplementary Figure 3) and urinary protein excretion (MD -0.10 g/24 h; 95% CI -0.24 to 0.04, *p* = 0.16; I^2^ = 93%) (Supplementary Figure 4), however, the studies suggested a higher risk of hyperkalemia (RR 2.43; 95% CI 1.14 to 5.19, *p* = 0.02; I^2^ = 68%) (Supplementary Figure 5).

## Discussion

Considering the probable risks of acute allograft injury, hyperkalemia, worsened anemia, and confusion or delay in the diagnosis of DGF and AR episodes are associated with early ACEI/ARB initiation post kidney transplant. Clinicians were troubled when they faced resistant hypertension and proteinuria in the early posttransplant period. To the best of our knowledge, guidelines or clinical practices have not reached an agreement to specify the optimal initiation time of ACEI/ARB in the early posttransplant period (Weir et al., 2015; Kramer et al., 2019). To improve clinical decision-making, several studies or RCTs have explored this urgent topic with extremely limited data. Two very early meta-analyses with limited sample sizes made pros and cons analyses that concluded controversial results by comparing ACEI/ARB with other antihypertensive drugs in KTRs. However, most KTRs included in their meta-analyses initiated ACEI/ARB after the third month posttransplant (Cross et al., 2009; Hiremath et al., 2007). Data from the early posttransplant period (within 3 months posttransplant) were relatively lacking and contradictory. Hence, we performed this meta-analysis to make conclusions about this argument through strict research review, data extraction and analysis.

Systemic and intrarenal RAAS activation was ubiquitous in KTRs (Kovarik et al., 2019; Antlanger et al., 2017). Goldblatt and colleagues concluded that renal ischemia precipitated the release of a pressor substance into the circulation. This pressor (and vasoconstrictive) substance was later found to be angiotensin II (ATII), which is generated by the release of renin by the ischemic kidney and activated by angiotensin-converting enzyme (ACE) (Goldblatt et al., 1934). The above studies provide a physiological background for RAAS blocker usage in KTRs. In addition to the advantages of lowering blood pressure, reducing proteinuria, lightening left ventricular hypertrophy (LVH) and treating posttransplant erythrocytosis, (Cruzado et al., 2008), ACEI/ARB was found to be preventive for interstitial fibrosis and tubular atrophy after kidney transplantation (Amer and Griffin, 2014; Sayin et al., 2017). Dragun and colleagues found that the angiotensin AT-1 receptor may be involved in vascular rejection, and affected patients might benefit from removal of AT1-receptor antibodies or from pharmacologic blockade of AT1 receptors, indicating a probable advantage in preventing acute rejection (Dragun et al., 2005). Moreover, Ahimastos and colleagues found that ACEI/ARB is believed to play a key role in vascular remodeling and the inflammation cascade (Ahimastos et al., 2005). Importantly, Lorenz’s study including 260 KTRs with an 80-day observation suggested that immediate posttransplant blockade of the RAAS did not increase the risk of DGF and was even favorable in shortening graft recovery time in such DGF cases; they explained that RAAS blockers could improve glomerular feedback mechanism disorder-associated GFR reduction via blockade of RAAS activation (Lorenz et al., 2004).

*Despite* these obvious clinical and physiological *advantages,* clinicians were still seriously concerned about adverse reactions of ACEI/ARB initiation within the first 3 months posttransplant, such as graft arterial stenosis and acute kidney injury, which might lead to irreversible graft injury and interfere with the judgment of graft function recovery. Kaleigh and colleagues suggested that the presence of bilateral renal artery stenosis was not associated with renin-angiotensin inhibitor use; in contrast, people with renal artery stenosis who received renin-angiotensin inhibitor treatment had significant benefits of blood pressure control, progression of renal disease, cardiovascular outcomes, and reduced all-cause mortality (Evans et al., 2014; Chrysochou et al., 2012). Lidija and colleagues compared serum creatinine at 0, 1, 3, 6, and 12 months posttransplant between two groups (ACEI/ARB initiation within 6 months and beyond 6 months posttransplant), and no significant difference was found at any estimate time, suggesting the safety of early initiation of ACEI/ARB in the posttransplant period (Orlić et al., 2013). In our analyses, ACEI/ARB initiated within the first month posttransplant did not increase short-term or long-term serum creatinine but improved short-term and long-term creatinine clearance. ACEI/ARB initiated in the first 1–3 months posttransplant reduced long-term serum creatinine but had no significant effect on short-term or long-term creatinine clearance. The effect on serum creatinine and creatinine clearance levels were not so coordinated but generally suggested its benefit on renal function recovery. At the same time, our data demonstrated that early initiation of ACEI/ARB did not increase the risk of DGF and AR but significantly reduced the risk of patient death and graft loss, which was consistent with previous studies (Jiang et al., 2018). Adverse events of ACEI/ARB use for KTRs, including hyperkalemia, anemia and GFR reduction, were reported, (Cruzado et al., 2008), however, our analyses indicated that early ACEI/ARB initiation did not lower hemoglobin levels or GFR but indeed significantly increased the risk of hyperkalemia. With inadequate participants and follow-up time, early ACEI/ARB initiation did not exhibit the advantage of proteinuria reduction. Last, no graft artery stenosis cases were reported in the included studies.

The above results concluded from our meta-analysis and reviewed literature suggested that early initiation of ACEI/ARB within the first 3 months posttransplant appears to be safe and with relatively good early graft function and long-term outcomes. In particular, recipients with good graft function recovery may particularly benefit from the early use of ACEI/ARB due to numerous positive effects but require frequent and careful monitoring of biochemical tests. In statistics, the inclusion of nonrandomized designs such as cohort studies could have increased our sample size and the generalizability of our findings. However, including such studies might have increased the risk of bias, thus making the interpretation of our findings difficult. Heterogeneity analyses found there were mild to moderate heterogeneities in patient death, graft loss, DGF, AR and hyperkalemia analyses, however, high level heterogeneities in serum creatinine and creatinine clearance were found, indirectly increasing the reliability of safety assessment of early initiation of ACEI/ARB. To conclude, further high-quality randomized trial evidence is warranted. Clinicians should weigh the risks and benefits of using ACEI/ARB with their patients on a case-by-case basis.

## Data Availability

The raw data supporting the conclusions of this article will be made available by the authors, without undue reservation.

## Abbreviations

RAAS, renin-angiotensin-aldosterone system; ACEI, angiotensin converting enzyme inhibitor; ARB, angiotensin receptor blocker; KT, kidney transplantation; KTRs, kidney transplant recipients; ESRD, end-stage renal disease; CKD, chronic kidney disease; RCT, randomized controlled trials; GFR, glomerular filtration rate; UPE, urinary protein excretion; DGF, delayed graft function; AR, acute rejection; POD, post-transplant day; MD, mean difference; ATII, angiotensin II; LVH, left ventricular hypertrophy. KDIGO, Kidney Disease: Improving Global Outcomes; AST, American Society of Transplantation; CENTRAL, Cochrane central register of controlled trials; RR, relative risk; CI, confidence interval; SD, standard deviation; SE, standard error.
