# Supplementary material for: Safety of ACEI/ARB use in the early (<3 months) post kidney transplant period: a systematic review and meta-analysis
Source: Front Pharmacol. 2024 Dec 11;15:1522558. doi: 10.3389/fphar.2024.1522558 (PMC11670068; doi:10.3389/fphar.2024.1522558)
Supplement: Supplementary file 1 [file DataSheet1.PDF]

Additional File 1 Search strategy

Search was conducted at 2023/12/31 by searching the PubMed, EMBASE and CENTRAL databases. Three additional studies were identified via citation.

Search strategy in PubMed from inception to 2023/12/31

#1 ((((((Angiotensin Converting Enzyme Inhibitors [Title/Abstract]) OR (ACE inhibitor[Title/Abstract])) OR (renin-angiotensin system[Title/Abstract])) OR (angiotensin II receptor blocker[Title/Abstract])) OR (renin-angiotensin-aldosterone system[Title/Abstract])) OR (ACEI[Title/Abstract])) OR (ARB[Title/Abstract])) OR (RAAS[Title/Abstract])

#2 (kidney transplant*[Title/Abstract]) OR (renal transplant*[Title/Abstract])

#3 #1 and #2

Search strategy in EMBASE from inception to 2023

#1 acei:ab,ti AND ([article]/lim OR [article in press]/lim) AND [humans]/lim AND [clinical study]/lim AND [embase]/lim AND [1966-2023]/py

#2 arb:ab,ti AND ([article]/lim OR [article in press]/lim) AND [humans]/lim AND [clinical study]/lim AND [embase]/lim AND [1966-2023]/py

#3 raas:ab,ti AND ([article]/lim OR [article in press]/lim) AND [humans]/lim AND [clinical study]/lim AND [embase]/lim AND [1966-2023]/py

#4'renin-angiotensin-aldosterone system':ab,ti AND ([article]/lim OR [article in press]/lim) AND [humans]/lim AND [clinical study]/lim AND [embase]/lim AND [1966-2023]/py

#5 'angiotensin ii receptor blocker':ab,ti AND ([article]/lim OR [article in press]/lim) AND [humans]/lim AND [clinical study]/lim AND [embase]/lim AND [1966-2023]/py

#6 'renin-angiotensin system':ab,ti AND ([article]/lim OR [article in press]/lim) AND [humans]/lim AND [clinical study]/lim AND [embase]/lim AND [1966-2023]/py

#7 'ace-inhibitor':ab,ti AND ([article]/lim OR [article in press]/lim) AND [humans]/lim AND [clinical study]/lim AND [embase]/lim AND [1966-2023]/py

#8 'angiotensin converting enzyme inhibitors':ab,ti AND ([article]/lim OR [article in press]/lim) AND [humans]/lim AND [clinical study]/lim AND [embase]/lim AND [1966-2023]/py

#9 #1 OR #2 OR #3 OR #4 OR #5 OR #6 OR #7 OR #8

#10 'kidney transplant*':ab,ti AND ([article]/lim OR [article in press]/lim) AND [humans]/lim AND [clinical study]/lim AND [embase]/lim AND [1966-2023]/py

#11 'renal transplant*':ab,ti AND ([article]/lim OR [article in press]/lim) AND [humans]/lim AND [clinical study]/lim AND [embase]/lim AND [1966-2023]/py

#12 #10 OR #11

#13 #9 AND #12

Search strategy in CENTRAL from inception to 2023.12.31

#1 (Angiotensin Converting Enzyme Inhibitors):ti,ab,kw OR ("ACE inhibitor"):ti,ab,kw OR ("renin angiotensin system"):ti,ab,kw OR ("angiotensin II-receptor blocker"):ti,ab,kw OR (renin-angiotensin-aldosterone system):ti,ab,kw

in Trials (Word variations have been searched)

#2 ("kidney transplantation"):ti,ab,kw OR (kidney transplantation):ti,ab,kw

in Trials (Word variations have been searched)
#3 #1 and #2

Four additional studies

1. Zhang, R., et al., Early inhibition of the renin-angiotensin system improves the long-term graft survival of single pediatric donor kidneys transplanted in adult recipients. Transpl Int, 2013. 26(6): p. 601-7.
2. Midtvedt, K., et al., Sustained improvement of renal graft function for two years in hypertensive renal transplant recipients treated with nifedipine as compared to lisinopril. Transplantation, 2001. 72(11): p. 1787-92.
3. Ibrahim, H.N., et al., Angiotensin II blockade in kidney transplant recipients. J Am Soc Nephrol, 2013. 24(2): p. 320-7.
4. Cockfield SM, Wilson S, Campbell PM, et al. Comparison of the effects of standard vs low-dose prolonged-release tacrolimus with or without ACEi/ARB on the histology and function of renal allografts. *American journal of transplantation : official journal of the American Society of Transplantation and the American Society of Transplant Surgeons.* 2019;19(6):1730-1744.

Study or Subgroup	log[Risk Ratio]	SE	ACEI/ARB Total	Non-ACEI/ARB Total	Weight	Risk Ratio IV, Fixed, 95% CI
Chatzikyrkou 2017	-0.5763	0.4765	142	114	17.0%	0.56 [0.22, 1.43]
Cockfield 2019	-1.9815	1.5071	142	137	1.7%	0.14 [0.01, 2.64]
Heinze 2006	-0.5621	0.2789	181	255	49.6%	0.57 [0.33, 0.98]
Ibrahim 2013	-0.1672	0.5323	77	76	13.6%	0.85 [0.30, 2.40]
Karsten 2001	1.6351	1.5409	76	78	1.6%	5.13 [0.25, 105.13]
Zhang 2019	-0.6554	0.4831	40	54	16.5%	0.52 [0.20, 1.34]
**Total (95% CI)**			**658**	**714**	**100.0%**	**0.60 [0.41, 0.88]**

Heterogeneity: Chi² = 3.45, df = 5 (P = 0.63); I² = 0%
Test for overall effect: Z = 2.62 (P = 0.009)

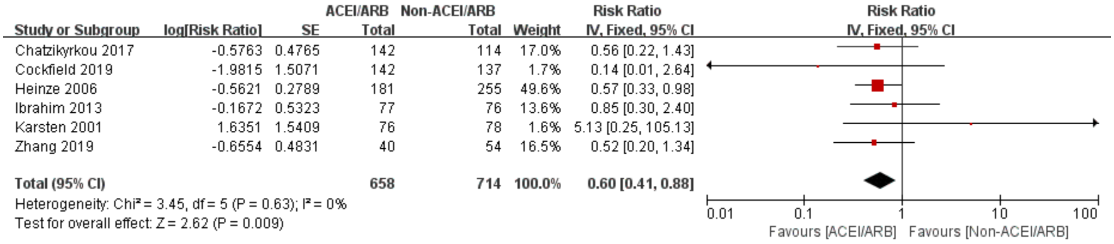

Additional figure 1 Forest plots depicting the risk ratios of patient death of early initiation of ACEI/ARB versus non-ACEI/ARB groups. CI, confidence interval.

Study or Subgroup	log[Risk Ratio]	SE	ACEI/ARB Total	Non-ACEI/ARB Total	Weight	Risk Ratio IV, Fixed, 95% CI
Chatzikyrkou 2017	-0.2196	0.5195	142	114	9.8%	0.80 [0.29, 2.22]
Cockfield 2019	-0.2182	0.5936	142	137	7.5%	0.80 [0.25, 2.57]
Heinze 2006	-0.7765	0.3261	181	255	25.0%	0.46 [0.24, 0.87]
Ibrahim 2013	-0.8095	0.4988	48	47	10.7%	0.45 [0.17, 1.18]
Zhang 2019	-0.6162	0.2377	40	54	47.0%	0.54 [0.34, 0.86]
**Total (95% CI)**			**553**	**607**	**100.0%**	**0.54 [0.40, 0.75]**

Heterogeneity: Chi² = 1.42, df = 4 (P = 0.84); I² = 0%
Test for overall effect: Z = 3.73 (P = 0.0002)

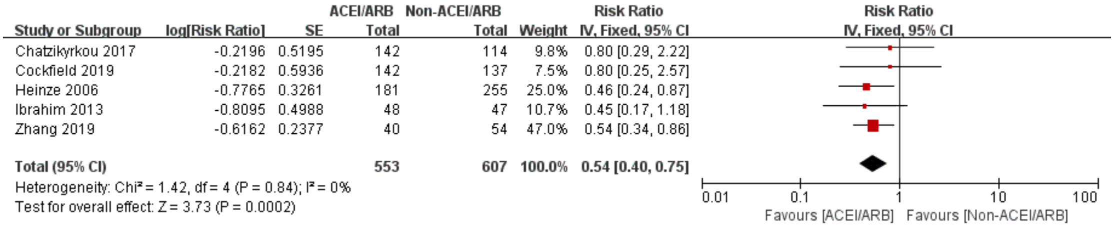

Additional figure 2 Forest plots depicting the risk ratios of graft loss of early initiation of ACEI/ARB versus non-ACEI/ARB groups. CI, confidence interval.

Study or Subgroup	ACEI/ARB Mean	SD	Total	Non-ACEI/ARB Mean	SD	Total	Weight	Mean Difference IV, Random, 95% CI
Formica Jr 2004	14.1	1	7	12.6	1.6	19	19.0%	1.50 [0.47, 2.53]
Formica Jr 2004	12.9	1.9	10	12.6	1.6	19	15.5%	0.30 [-1.08, 1.68]
Karsten 2001	12.5	1.7	54	13.8	2	69	23.0%	-1.30 [-1.95, -0.65]
Richard 2006	12.2	2.2	17	13.9	1.3	10	16.1%	-1.70 [-3.02, -0.38]
Suwelack 2003	12.9	0.4	48	13.2	0.4	48	26.4%	-0.30 [-0.46, -0.14]
**Total (95% CI)**			**136**			**165**	**100.0%**	**-0.32 [-1.16, 0.52]**

Heterogeneity: Tau² = 0.69; Chi² = 25.57, df = 4 (P < 0.0001); I² = 84%
Test for overall effect: Z = 0.74 (P = 0.46)

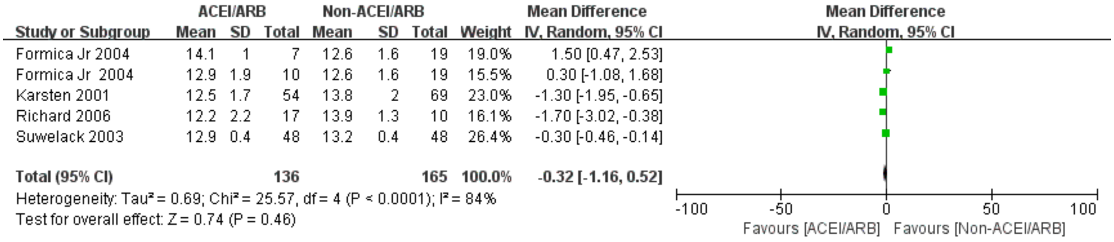

Additional figure 3 Forest plots of mean hemoglobin differences between the ACEI/ARB and non-ACEI/ARB groups. CI, confidence interval.

Study or Subgroup	ACEI/ARB Mean	ACEI/ARB SD	ACEI/ARB Total	Non-ACEI/ARB Mean	Non-ACEI/ARB SD	Non-ACEI/ARB Total	Weight	Mean Difference IV, Random, 95% CI
Chatzikyrkou 2017	0.17	0.3	142	0.14	0.33	114	30.0%	0.03 [-0.05, 0.11]
Hausberg 1999	0.4	0.1	35	0.6	0.2	35	30.3%	-0.20 [-0.27, -0.13]
Lorenz 2004	0.97	1.2	116	1.74	2.6	144	6.6%	-0.77 [-1.25, -0.29]
Suwelack 2003	0.31	0.03	48	0.3	0.04	48	33.1%	0.01 [-0.00, 0.02]
**Total (95% CI)**			**341**			**341**	**100.0%**	**-0.10 [-0.24, 0.04]**

Heterogeneity: Tau² = 0.01; Chi² = 40.31, df = 3 (P < 0.00001); I² = 93%

Test for overall effect: Z = 1.42 (P = 0.16)

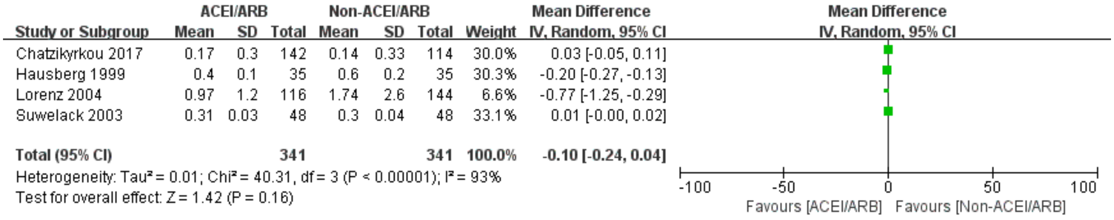

Additional figure 4 Forest plots of mean urinary protein excretion differences between the ACEI/ARB and non-ACEI/ARB groups. CI, confidence interval.

Study or Subgroup	ACEI/ARB Events	ACEI/ARB Total	Non-ACEI/ARB Events	Non-ACEI/ARB Total	Weight	Risk Ratio M-H, Random, 95% CI
Cockfield 2019	52	142	42	137	34.4%	1.19 [0.86, 1.66]
Glicklich 2011	2	13	1	11	8.6%	1.69 [0.18, 16.25]
Ibrahim 2013	17	77	5	76	23.4%	3.36 [1.30, 8.64]
Karsten 2001	22	54	8	69	27.5%	3.51 [1.70, 7.27]
Richard 2006	6	29	0	27	6.0%	12.13 [0.72, 205.60]
**Total (95% CI)**		**315**		**320**	**100.0%**	**2.43 [1.14, 5.19]**
Total events	99		56			

Heterogeneity: Tau² = 0.41; Chi² = 12.44, df = 4 (P = 0.01); I² = 68%

Test for overall effect: Z = 2.29 (P = 0.02)

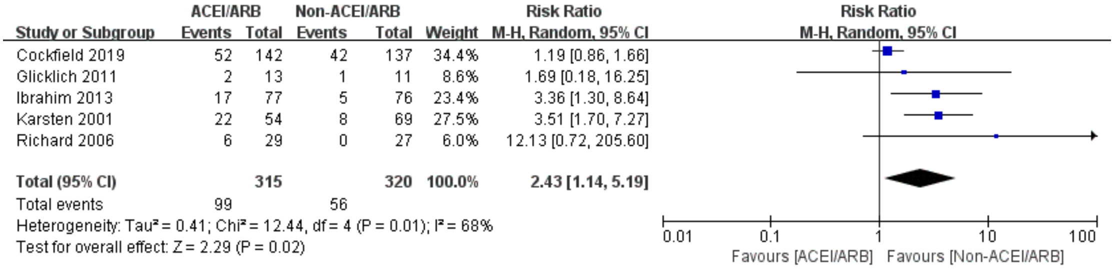

Additional figure 5 Forest plots depicting the risk ratios of hyperkalemia of early initiation of ACEI/ARB versus non-ACEI/ARB groups. CI, confidence interval.
